# Radial neck fracture or Monteggia equivalent lesion: delayed radial head subluxation in an adolescent and review of literature

**DOI:** 10.1186/s12891-020-03315-0

**Published:** 2020-05-06

**Authors:** Lujie Xu, Wensong Ye

**Affiliations:** Department of Orthopaedics, Children’s Hospital, Zhejiang University School of Medicine, 14th Floor, Inpatient Building, No. 3333 Binsheng Road, Hangzhou Zhejiang, People’s Republic of China

**Keywords:** Monteggia fracture, Monteggia equivalent lesion, Radial neck fracture, Radiocapitellar dislocation, Adolescent, Children

## Abstract

**Background:**

Monteggia equivalent lesion represents a series of combined elbow and forearm injuries that resemble typical Monteggia fracture either in presentation or mechanism. The term has gradually evolved since its introduction, as sporadic case reports continued to complement it. The aim of this study was to present a furthermore type of that lesion which no previous study had reported and arouse pediatric orthopedists’ additional awareness of it.

**Case presentation:**

A 11-year-old girl, whose injury pattern initially appeared to be a mild radial neck fracture with undisplaced proximal ulnar fracture, and without radial head dislocation, was treated with closed reduction and long-arm splint immobilization. Acceptable results were acquired at first-week follow-up, yet dramatic changes turned up 2 weeks later when the dislocated radial head was found. A further reduction to the fracture and joint site only resulted in a subluxated and incongruous radiocapitellar joint on the three-dimensional computed tomography (3D-CT). Then a definitive operation was performed, which involved a Boyd incision, correction of radial head tilting, opening wedge osteotomy of the proximal ulna and proper fixation respectively. And acceptable results were achieved 1 year later.

**Conclusions:**

This case, with occult proximal ulna fracture, angulated radial neck fracture, subsequent radiocapitellar dislocation, and articular incongruity, was deemed as a rare Monteggia type-one equivalent fracture-dislocation variant rather than an ordinary radial neck fracture and it facilitates further understanding and management of the Monteggia fracture.

## Background

Monteggia fracture, named after Giovanni Monteggia in the nineteenth century and well described and classified by Dr. Bado decades ago, involves ulnar fracture and concomitant dislocation of the radial head. The term “fracture” has gradually been replaced by those like “lesion”, “fracture-dislocation”, “injury” in literature, stressing the importance of radiocapitellar joint and reflecting an increasing awareness of the complexity regarding its manifestation and mechanism among pediatric orthopedists.

Apart from the four distinctive patterns, the so-called concept of “Monteggia equivalent lesion/variant” has since been considerably expanded with decades of sporadic reports. A quite number of these types tend to be misdiagnosed or neglected due to the occult presentation of radiocapitellar joint or bowing ulna on radiographs, especially in pediatric patients when immature epiphysis interferes with judgment.

The case we presented represents one of the tricky Monteggia equivalent fracture-dislocation variants that no previous study has ever reported. Pediatric orthopedists’ additional awareness of this variant facilitates further understanding and management of the Monteggia fracture.

## Case presentation

**Fig. 1:**
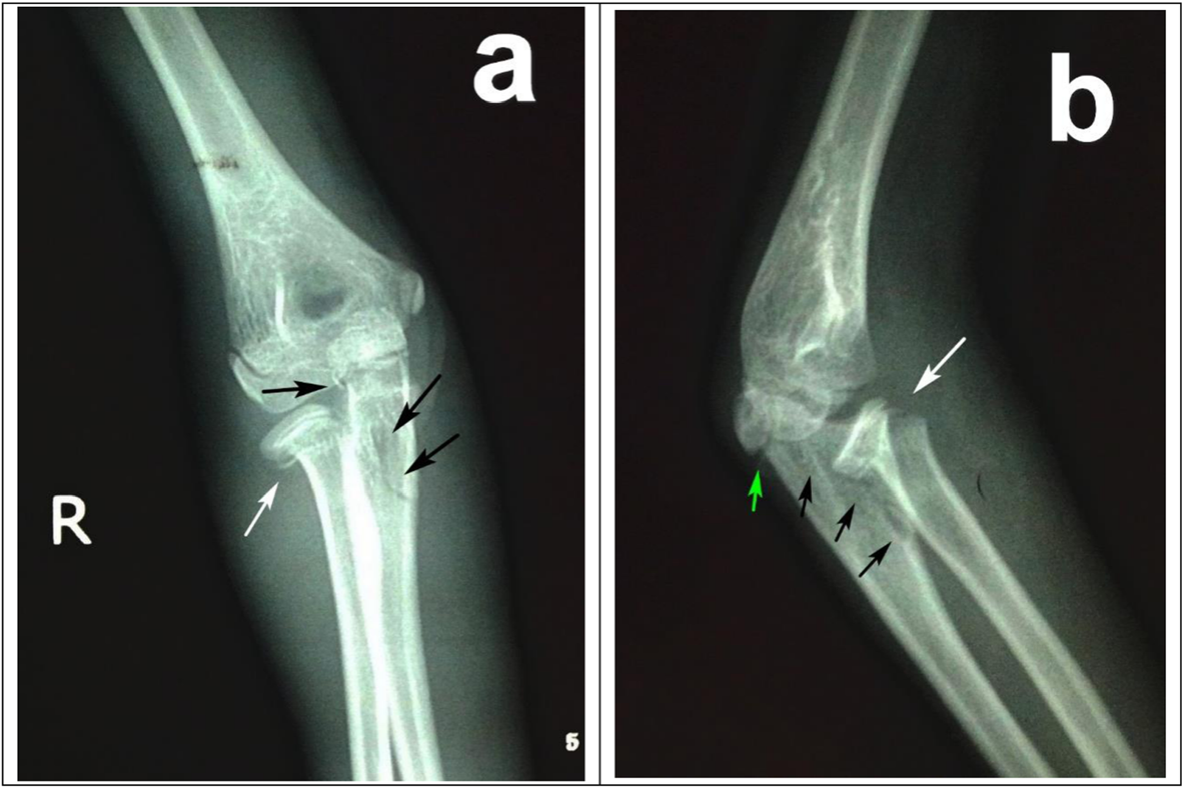
**a** Initial AP X-ray showed Jeffery type-1 radial neck fracture, indicating a lateral angulation of the radial head (White arrow) as a result of valgus stress, and proximal ulnar fracture (Black arrow). **b** Initial lateral X-ray showed anteriorly angulated radial neck fracture with intact radiocapitellar joint (White arrow) and proximal ulna fracture with fracture line going through olecranon epiphyseal plate (Green arrow) and potential anterior bowing (Black arrow)

**Fig. 2:**
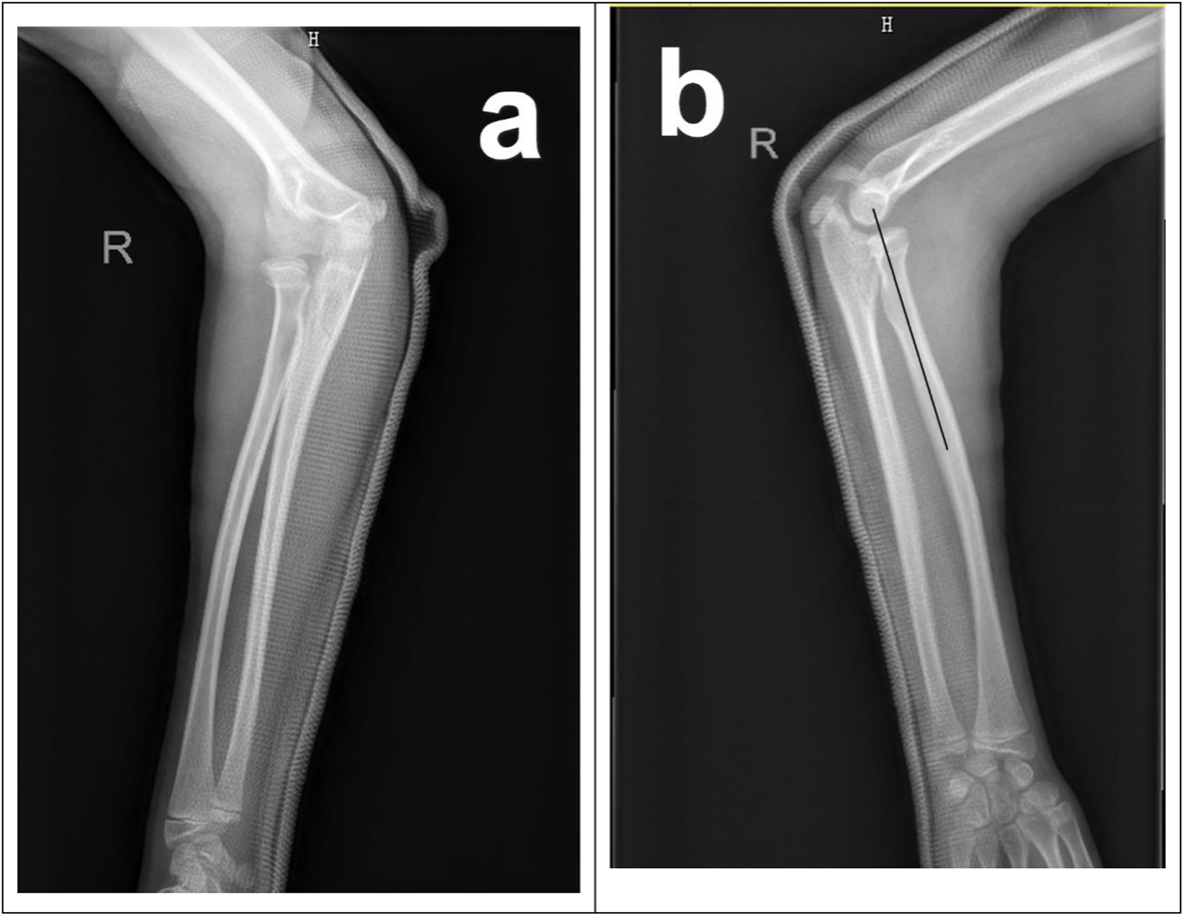
**a** AP X-rays showing proper fracture and joint position after initial closed reduction. **b** Lateral X-rays showing proper fracture and joint position after initial closed reduction. Black line showing axis of radius, the extension of which went right through the capitellum

**Fig. 3:**
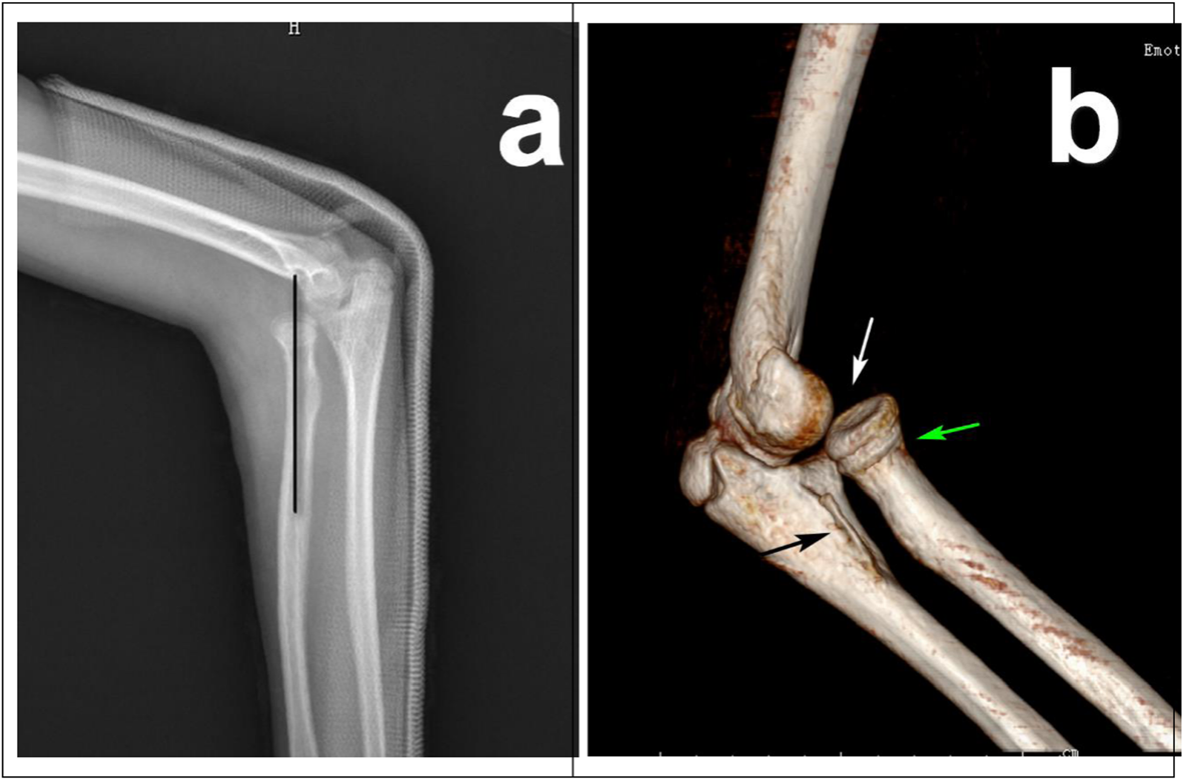
**a** Three-week post-traumatic lateral X-ray showed anterior dislocation of radial head. Black line showing axis of radius, the extension of which didn’t go through the capitellum. **b** 3D- CT after secondary closed reduction showed subluxation and incongruity of the radiocapitellar joint. White arrow: the radiocapitellar joint; Black arrow: unhealed and potential anterior bowing of proximal ulnar fracture. Green arrow: unhealed and angulated radial neck fracture

**Fig. 4:**
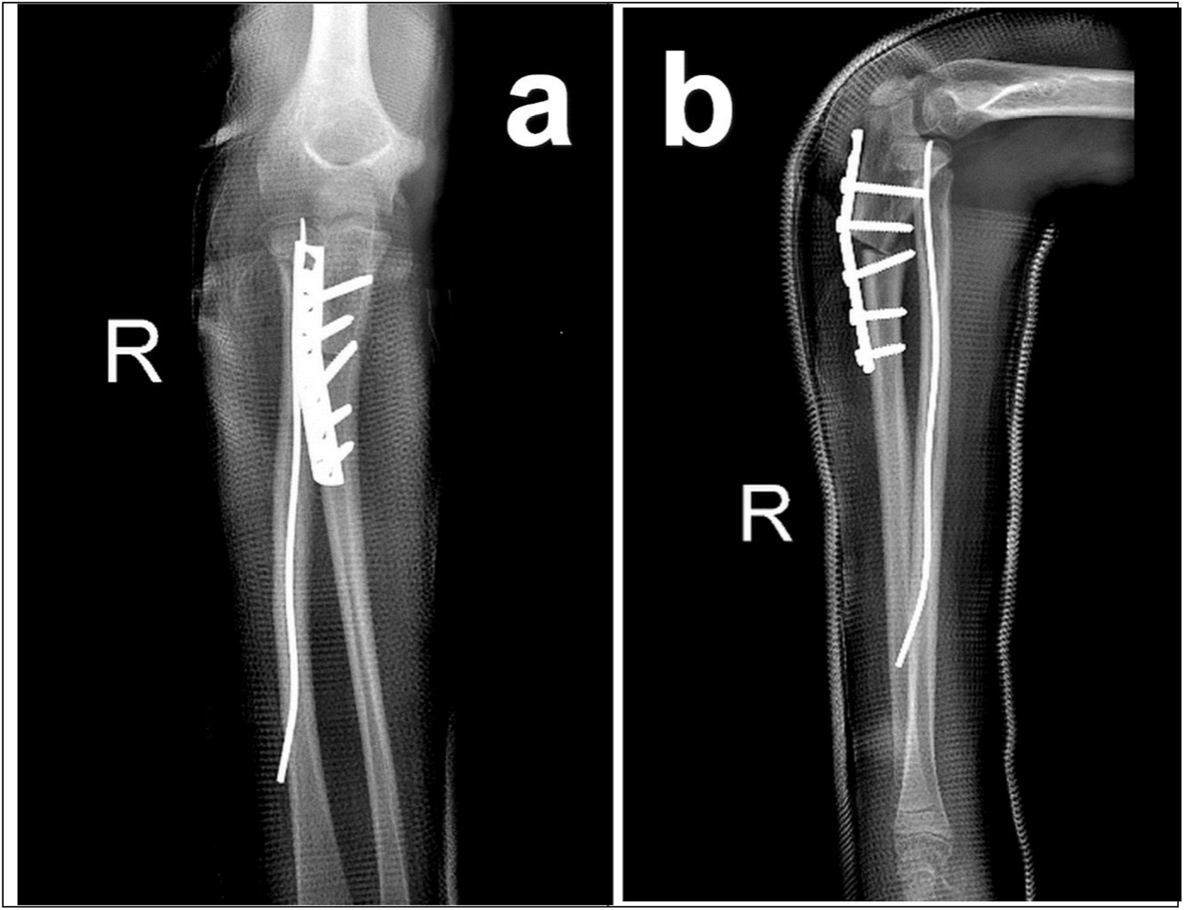
**a** Post-operational AP X-rays showing fixation of radial neck and proximal ulna with elastic intramedullary nail and plate/ screws. **b** Lateral X-rays showing fixation of radial neck and proximal ulna with elastic intramedullary nail and plate/ screws

**Fig. 5:**
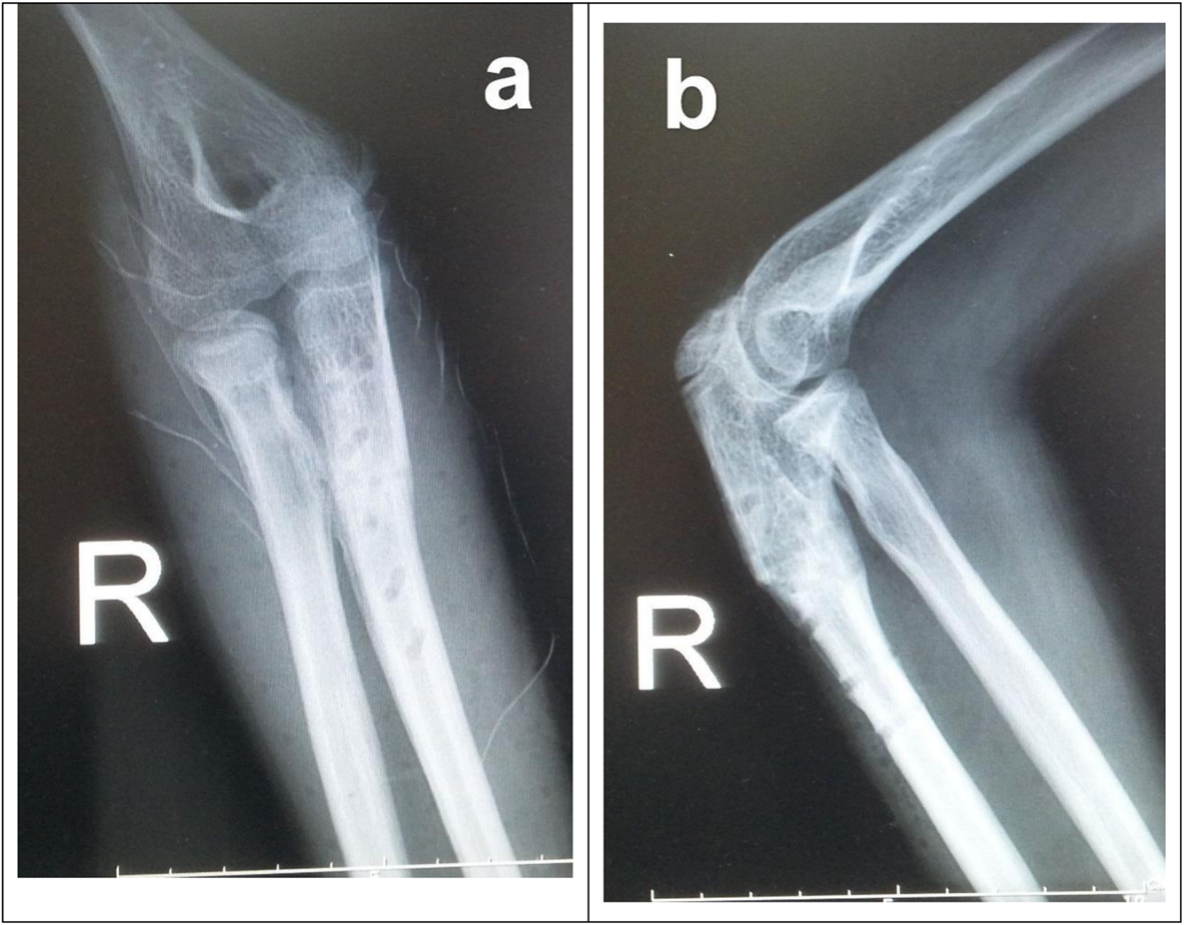
**a** Final AP X-rays at one-year follow-up showing bony union in the radial and ulnar osteotomy sites with excellent radiocapitellar congruity and proximal ulnar shaping. **b** Final lateral X-rays at one-year follow-up showing bony union in the radial and ulnar osteotomy sites with excellent radiocapitellar congruity and proximal ulnar shaping

An 11-year-old right-hand dominated girl initially presented at our clinic with X-rays taken at a community clinic. It was 1 day after she fell off a chair with the affected upper limb stretched out for a book on a shelf. The X-rays (Fig. 1) appeared to show a Jeffery type-1 radial neck fracture with undisplaced proximal ulnar fracture and an “intact” radiocapitellar joint. With radial neck fracture at the epiphyseal site, radial head tilted less than 30 degrees, laterally displaced less than 2 mm, this case was treated as a “mild” one by the initial physician with close reduction (Patterson’s Maneuver) and long-arm splint immobilization. Radiological results turned out acceptable in the first-week follow-up (Fig. 2). Dramatic changes had not shown up until 3 weeks post-trauma, when anterior radial head dislocation was found (Fig. 3a). One more close reduction and cast immobilization were performed. Three days later, the radial head dislocated again and the elbow movement restriction was found. Then a 3D-CT was ordered (Fig. 3b). The operation was performed, which comprised scar tissue releasing, humeroradial joint reduction, radial neck de-angulation osteotomy and fixation with an elastic intramedullary nail, proximal dorsal ulnar opening-wedge osteotomy, and fixation with pre-angulated plate and screws. A long arm splint was used (Fig. 4). The splint was taken off 2 weeks postoperatively and early functional training was ordered. The radial neck fracture and the ulnar osteotomy reached bony union at 10 weeks postoperatively and the internal fixation was taken off 1 week later. The patient was followed for 1 year after surgery. The last X-ray was recorded (Fig. 5). The affected elbow and forearm gained full range flexion, five degrees extension loss and full supination, 40 degrees pronation. The carrying angle remained the same as the contralateral side and no neurovascular defects resided. The patient returns to normal sports.

## Discussion and conclusion

Early in 1814, Monteggia [1] first named the fracture-dislocation pattern that includes ulnar fracture and concomitant radial head dislocation as Monteggia fracture. And Bado [2] in 1967 proposed the classification system, now widely used, and with it the term “Monteggia equivalent lesion” as well as the four common types based on the direction of radial head dislocation. Five equivalent lesions were described in his classification. A more comprehensive category model was suggested by Letts [3] in 1985, stressing the importance to notice ulnar bowing specifically in pediatric patients. In 1990, Olney [4] formally developed one typically for childhood Monteggia lesions. Three Monteggia equivalent lesions were described in this classification and typically for those sporadic cases which resemble the Monteggia mechanism, but could not categorize into the common types. However, the established equivalent lesions in those classifications failed to cover the pattern this case presented, involving delayed radiocapitellar dislocation and prior radial neck fracture.

Up to date, through literature in Scientific Citation Index, we found a few authors had referred to the terms of “equivalent lesion/injury/fracture-dislocation” [5–19], some of which met the criteria of Bado or Olney. (Table 1–2) All of the presented cases involved the injury of both radius and ulna, especially the region around the radiocapitellar joint, reflecting a rationale expressed by Bado that morphological change of ulna incurs proximal radial and/or radiocapitellar joint injury [2]. Sur [11], Reina [13], Sirois [18] and Heinrich [19], reported type-3 equivalent mechanism of posterior bowing or angulation of ulna, leading to radial shaft posterior translocation and proximal radius/ radiaocapitellar joint lesion. And some rest others [7–10, 12, 14, 15] reported type-1 equivalent cases of anterior bowing or angulation of ulna, resulting in radial shaft anterior translocation and similarly proximal radius/ radiaocapitellar joint lesion, as listed in Table 1. Due to photographic and print quality, some dated reports could not properly categorize [5, 6].

Most of the cases demonstrated acute dislocation of radiocapitellar joint or acute radial shaft and neck separation but intact joint while only rest two showed some sort of chronic change of proximal radius region [11, 19]. As Weisman [20] suggested, approximately 2% of Monteggia lesions present with a reduced radial head, the dislocation occurring after the initial evaluation. And Sur [11]‘s case suggested delayed dislocation of radiocapitellar joint aside, delayed migration of radial shaft from the neck towards ulnar bowing direction could also occur.

Among those cases, the one Ruchelsman [8] presented, of an injury pattern involving anterior dislocation of the radial head and fractures of the olecranon and radial neck, which he concluded into a rare type of Monteggia equivalent lesion variant, mostly resemble what we reported. Yet five reasons distinguished our case from it. First, anterior dislocation of the radiocapitellar joint existed on initial X-ray but neglected in his case, whereas in our case radiographic results suggest a radial neck fracture with intact joint initially. Second, the proximal ulnar was injured as a pattern of hairline fracture [Fig. 1, black arrow] instead of olecranon fracture in his case. Third, it was an adolescent instead of a preschool child that we presented. The age matters much for pediatric orthopedic patients so the two cases could be of different mechanisms and justify varied management. Fourth, in our case, elastic nailing was chosen to correct the tilt of the radial neck, which was introduced by Faundez [21] and Metaizeau [22] in their cases respectively. Opening wedge ulnar osteotomy, plate and screws fixing were applied to stabilize the radiocapitellar joint, to which was referred by Hirayama [23]. Yet in Ruchelsman’s case, radial neck fracture was fixed by 2 k-wires and annular ligament was reconstructed, while ulna remained unoperated. Finally, fairly good outcomes were achieved in our case both clinically and radiologically at the one-year follow-up. Though clinical results were good in Ruchelsman’s case for half a year, however, final X-ray results revealed proximal radial notch, enlarged radial head and probable early heterotopic ossification. Long term outcomes may not be optimistic. From the above, this case was considered as a non-reported rare pattern of Monteggia type-1 equivalent lesion.

Due to the rarity, the mechanism for this lesion have to be inferred from those resembling cases. As for pediatric radial neck fracture, the classical mechanism was introduced by Jeffery as the compression force on the radial side and the associated traction lesions on the ulnar aspect [24]. While the mechanism of type-1 monteggia fracture was expressed by Bado as a forearm hyper pronation, and the ulnar fracture or bowing towards the proximal radius, causing the radial head dislocation [2]. Tompkins added to it later a mechanism of elbow hyperextension, which attributed the anterior dislocation of radial head to contracture of bicep muscle when hyperextended [25]. Plastic deformity of the ulna also played a great role in radial head dislocation, as Kay reported [26]. Recently three-dimensional analysis suggested the dislocation as a result of forearm pronation and ulnar plastic bowing [27]. As for this case, taking into account the prior and following appearance, the mechanism should be considered a combination, that initial compression and associated traction force caused the radial neck and proximal ulnar fracture; hyperextension led to anterior bowing ulna and contracture of bicep muscle which angulated the fractured neck. And the potential bowing ulna with soft tissue lesion around the radial neck made it possible for secondary dislocation of the radiocapitellar joint.

Specifically in this case, the initial X-ray (Fig. 1) would hardly suggest the involvement of the annular ligament and the radiocapitellar capsule. The anterior-posterior (AP) view (Fig. 1a) revealed a typical Jeffery [24] type-1 radial neck fracture, indicating a lateral angulation of the radial head (White arrow) as a result of valgus stress, which could also result in the proximal ulnar fracture (Black arrow), though not quite obvious. Whereas the lateral view (Fig. 1b) suggested something else. The radial neck fracture was also anteriorly angulated, though joint intact, (White arrow), suggesting an anterior force from potential bowing ulna (Black arrow), which was also a Monteggia mechanism proposed by Bado. On both considerations, closed reduction turned to be the first choice and the radiologic results suggested it did work at that time. Delayed radiocapitellar subluxation might imply a rupture of the annular ligament or collateral ligament due to primary trauma, which had been identified by the following surgery. And the lurking anterior bowing ulna might provide a persistent force on the radial head. Also, relative insufficient immobilization of a single back splint and propensity of the young patient to be restless added the inclination for radial head dislocation. Then incongruity of the radiocapitellar joint, lurking bowing of proximal ulna and scar tissue around radial neck prevented viability of second closed reduction (Fig. 3b).

On considerations of the above, we might take some management notes for pediatric radial neck fracture with proximal ulna involved. Initial closed reduction should be properly handled with a sufficiently immobilized cast or double splints. The magnetic resonance imaging (MRI) or elbow arthrography are better ordered when plain X-ray reveals tricky manifestation. Finding potential ulnar bowing and annular ligament rupture will facilitate predicting the tardy dislocation of the radiocapitellar joint. Once a delayed radial head dislocation developed, especially combined with humeroradial joint incongruity and potential ulnar bowing, indication for surgery could be explicitly clear. Radial neck tilt ought to be corrected, to avoid incongruity of the radiocapitellar joint even if the joint was reduced. A process to use a K-wire to lever the radial neck and intramedullary nail to stabilize it was suggested by Ceroni [28] to acquire that end, which was adopted in our case. Whether to reconstruct the annular ligament was a little controversial in the circumstance. In this case, the capsule was appropriately tightened to provide stability whereas the annular ligament was left aside. Further radiocapitellar stability was obtained from ulnar bony morphology. Correction of ulnar bowing by opening wedge osteotomy had been exemplified by Hirayama [23] and Exner [29] respectively as an effective way. Gradual angulation using external fixation as Exner introduced proved more effective in marked dislocated cases. To avoid the prolonged pin-holes caring and infection risk, we performed one stage ulnar osteotomy- the Hirayama way. The Boyd [30] incision was appropriate for adequate exposure of both proximal radius and ulna. Anyway, however, aside from bizarre appearances, tricky and prolonged management and recovery process, most reported cases including this one revealed good mid-term outcomes.

Pediatric Monteggia equivalent lesion remains a tricky condition to distinguish due to occult presentation on plain radiographs of unclear epiphysis-comprised radiocapitellar joint or underlying hard-to-discern bowing ulna. Diagnostic and treating dilemma regarding it was abundant in the literature. However, this report presented a previously undescribed type-I Monteggia equivalent lesion with a prior radial neck fracture, late radiocapitellar dislocation, and proximal ulna crack. The inferred mechanism and managing procedure, though unable to be a guideline due to the low-evidence nature of this study, was still instructive for those dealing with pediatric elbows. The authors suggest ordering the MRI or elbow arthrography when plain X-ray reveals tricky manifestation in those radial neck fractures with proximal ulna involved. Finding potential ulnar bowing and annular ligament rupture will facilitate predicting the tardy dislocation of the radiocapitellar joint. Though initial closed reduction is still optimal, close follow-up is firmly required and proper surgery procedure is needed when dislocation of redial head turns chronic.

## Data Availability

Whole data and material needed to support our findings were included in the paper and available for publication. Confidential patient data was not shared.

## Abbreviations

3D-CT: Three-dimensional computed tomography
Fig.: Fig.
AP: Anterior-posterior
MRI: Magnetic resonance imaging
